# Find-DLB: a naturalistic cohort of patients presenting with clinical features of dementia with Lewy bodies to a specialized cognitive clinic

**DOI:** 10.1007/s41999-025-01372-z

**Published:** 2025-12-08

**Authors:** Stephanie Gravett, Sara Garcia-Ptacek, Anna Rennie, Nenad Bogdanovic, Alexandre Bonnard, Tobias Granberg, Agneta Nordberg, Vesna Jelic, Daniel Ferreira

**Affiliations:** 1 Division of Clinical Geriatrics, Center for Alzheimer Research, Department of Neurobiology, Care Sciences and Society, Karolinska Institutet, Stockholm, Sweden; 2 Theme Women’s Health and Allied Health Professionals, Karolinska University Hospital, Stockholm, Sweden; 3 Theme Inflammation and Aging, Karolinska University Hospital, Stockholm, Sweden; 4 Department of Neuroradiology, Karolinska University Hospital, Stockholm, Sweden; 5 Department of Clinical Neuroscience, Karolinska Institutet, Stockholm, Sweden; 6 Facultad de Ciencias de la Salud, Universidad Fernando Pessoa Canarias, Las Palmas de Gran Canaria, Spain

**Keywords:** DLB, PDD, Synucleinopathy, Lewy body dementia, Neuropsychology, Biomarkers

## Abstract

**Purpose:**

Dementia with Lewy bodies (DLB) is a common neurodegenerative disorder, yet difficult to diagnose. Carefully selected research cohorts may not represent the clinical reality. We aimed to characterize a naturalistic cohort of patients with clinical features of DLB, reporting their final diagnosis, clinical features, and cognitive profile.

**Methods:**

Patients were recruited from a specialized cognitive clinic. Data from patient visits such as core clinical features, biomarkers, cognitive screening, and neuropsychological assessment were collected from health records. We used normative data to assess distribution of impairment in patients with DLB and Parkinson’s disease (PD) with and without dementia.

**Results:**

A total of 143 patients were included in the cohort. Following specialized dementia evaluation, 88 patients fulfilled clinical criteria for DLB, 35 patients for PD with dementia (PDD), 14 had mild cognitive impairment (MCI), and the remaining 6 patients had other types of dementia. Parkinsonism was the most common core clinical feature (87%), followed by visual hallucinations (65%), cognitive fluctuations (52%) and, lastly, probable REM sleep behavior disorder (RBD, 47%). A majority of DLB patients had cognitive impairment on visuospatial constructive, attentional and executive tasks, and visual memory.

**Conclusions:**

The differential diagnosis of DLB may be difficult within a clinical context because other cognitive disorders frequently present with core features of DLB. The cognitive profile and frequency of core clinical features in the DLB group were generally in line with previous reports. Probable RBD was lower than in other cohorts, which may reflect challenges in collecting this information in a clinical interview.

**Supplementary Information:**

The online version contains supplementary material available at 10.1007/s41999-025-01372-z.

## Key summary points

**Aim:**

We aimed to characterize a naturalistic cohort of patients with symptoms of Dementia with Lewy Bodies (DLB).

**Findings:**

While the majority (88/143) received a clinical diagnosis of DLB, 35 patients received a diagnosis of Parkinson’s disease with dementia, and 20 patients received a diagnosis of other dementia or mild cognitive impairment. Parkinsonism was the most frequent core feature, and DLB patients commonly showed impairment in visuospatial, attentional, executive, and visual memory tasks.

**Message:**

The differential diagnosis of DLB is challenging in a clinical setting due to overlapping features and requires careful review of clinical features and biomarkers.

**Supplementary Information:**

The online version contains supplementary material available at 10.1007/s41999-025-01372-z.

## Background

Dementia with Lewy bodies (DLB) is a common neurodegenerative cause of dementia, characterized by core clinical features of parkinsonism, visual hallucinations, cognitive fluctuations, and REM sleep behavior disorder (RBD) [1]. A previous systematic review reported DLB to account for about 5% of dementia cases, although prevalence values ranged from 0.3 to 24.4% [2]. This wide range highlights differences between cohorts and disparities in how DLB is diagnosed and participants recruited to studies across the globe. Indeed, DLB is known to be underdiagnosed, and differences in prevalence rates in specialized cognitive clinics may be influenced by factors such as sensitivity to core clinical features, education or training of clinical staff, and research focus of specific centers [3, 4]. One reason for the diagnostic challenges is the significant overlap between DLB and other disorders such as Alzheimer’s disease (AD), Parkinson’s disease with dementia (PDD), and cerebrovascular disease. While clinical presentations may converge, there is also pathological overlap. Around 50% of DLB patients have concomitant AD pathology [5–7]. This concomitant AD pathology and positivity of AD biomarkers confound the diagnostic accuracy in DLB [8]. Additionally, there are challenges differentiating DLB from PDD, especially when cognitive impairment progresses rapidly and occurs early during Parkinson’s disease (PD).

Cognitive profiling with neuropsychological assessment is an important diagnostic tool within dementia evaluation to differentiate the level and type of cognitive impairment. The cognitive profile in DLB generally constitutes impairment in visual processing, attention, and executive functions with relative sparing of memory and naming, differentiating it from AD [1]. The differences in the cognitive profile of DLB versus PDD have been partially explored, but results are not conclusive [9, 10].

While several DLB cohorts have been well characterized, these cohorts were primarily research oriented [11–13], so that study participants may be different from those presenting at specialized cognitive clinics. There are fewer publications of naturalistic clinical cohorts describing DLB patients [14], and the clinical journey of patients presenting to the cognitive clinic with a suspicion of DLB is generally not reported and largely unknown. Investigating this patient group is thus relevant to capturing a more complete picture of DLB and understand potential bias in the reported profiles of patients eventually being recruited to well-characterized research-oriented cohorts.

The current study describes Find-DLB, a naturalistic clinical cohort of patients presenting with clinical features of DLB to a specialized cognitive clinic. The goal was to present a realistic view of the diagnostic flow, which may help increase the knowledge on the day-to-day challenges clinicians face when differentiating DLB from other neurodegenerative disorders, non-degenerative causes of cognitive impairment such as psychiatric illnesses, and normal aging. We aimed to present the baseline demographic, clinical, biomarker, and neuropsychological data of the original cohort as well as stratified by diagnostic group after classification in naturalistic clinical rounds.

## Methods

### Study setting

The Cognitive clinic at Karolinska University Hospital Huddinge is a specialist unit for cognitive disorders. Patients are referred from their primary care facility or occasionally from other specialist units on suspicion of cognitive disorder. Approximately, 600 referrals are accepted each year. Both early-onset (< 65y) and late-onset (> 65y) patients are evaluated at the clinic. Patients > 65y are usually from the close geographical area, while < 65y patients may be referred from the entire Stockholm Region, which comprises several municipalities over an area of approximately 6500 square km, with a population of 2 450 000 in 2023 [15]. Second opinions may be referred from other Swedish regions across the country as well.

**Fig. 1:**
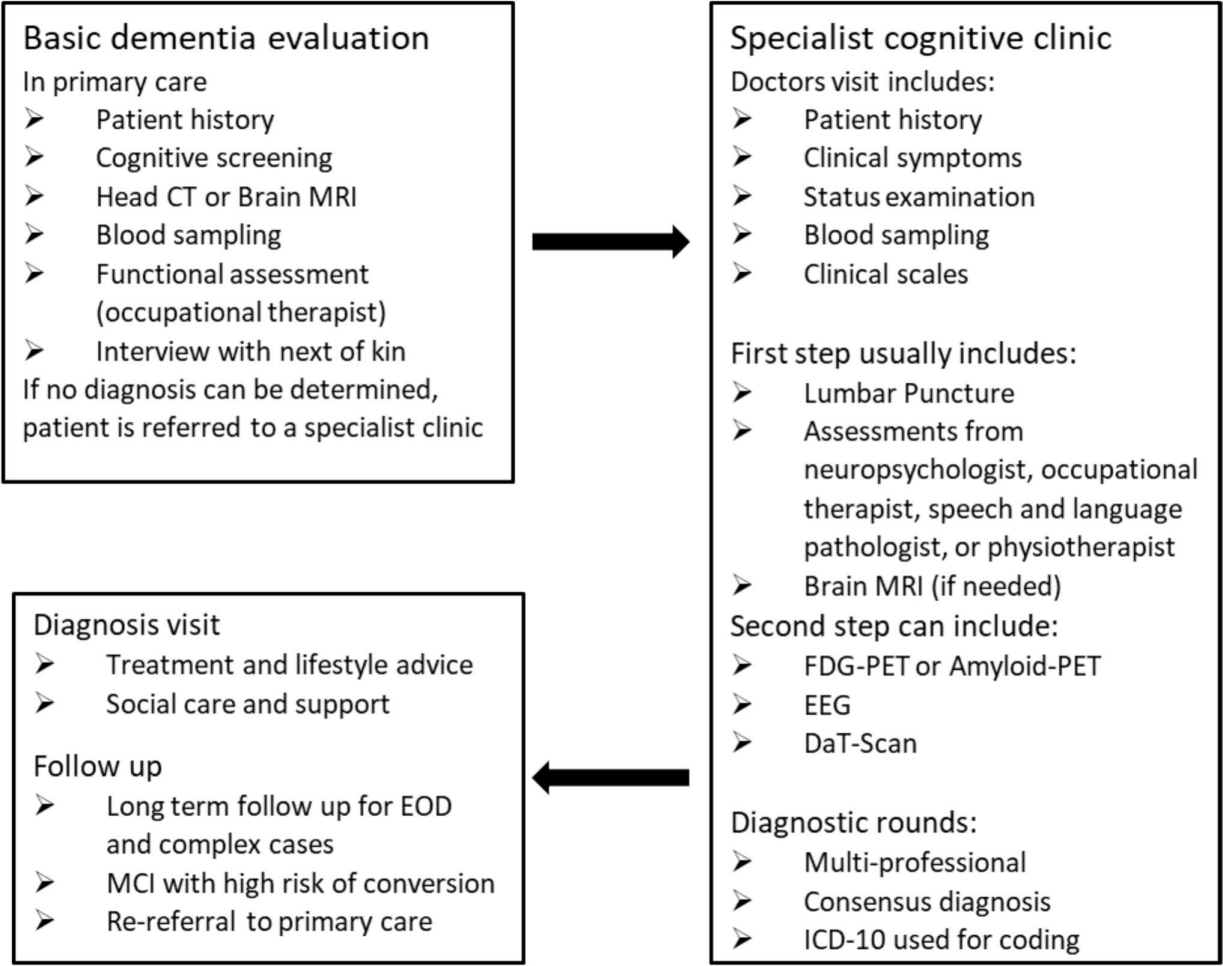
Flowchart of the evaluation process at the Karolinska University Hospital Huddinge Cognitive Clinic. *CT* computer tomography, *MRI* magnetic resonance imaging, *FDG* fluorodeoxyglucose, *PET* positron emission tomography, *EEG* electroencephalography, *DaT-Scan* dopamine transporter scan, *EOD* early-onset dementia, *MCI* mild cognitive impairment (referring to MCI with high risk of conversion to dementia)

Prior to referral, most patients had undergone a basic dementia evaluation in primary care, often including a computer tomography (CT) scan or magnetic resonance imaging (MRI) of the brain, blood sampling, assessment of activities of daily living (ADL), as well as cognitive screening with the Mini Mental State Examination (MMSE), Montreal Cognitive Assessment (MoCA), or the Rowland Universal Dementia Assessment Scale (RUDAS) [16–18]. As reported in Fig. 1, the specialist dementia evaluation can include a range of clinical assessments, neuroimaging modalities, and lumbar puncture depending on clinical suspicion. Usually, clinical assessments and lumbar puncture comprise the first step of the diagnostic workup at the specialized cognitive clinic. Additional tests can be added thereafter in the second step if needed. The aim is to complete the specialist diagnostic workup in a maximum of 3 months according to Swedish praxis.

Diagnoses are determined at multi-professional rounds comprising four to ten people, including physician, neuropsychologist, nurse, occupational therapist, and speech and language pathologist. All relevant information from the examinations is presented and diagnosis is determined by consensus principle. The ICD-10 is used for diagnostic coding. Patients are often subject to follow-up at the clinic, and they may therefore repeat evaluations over time. Assessments might be added by the responsible physician during follow-up to help differentiate the diagnosis in complex cases. Diagnoses can be revised at an additional diagnostic round whenever needed. The full process is illustrated in Fig. 1.

### Participants

Find-DLB was started in 2020 with the main goal of improving detection of DLB in specialized cognitive clinics in Stockholm, Sweden. Find-DLB includes both a retrospective and a prospective approach, including retrospective patients up to 2020, and recruiting patients prospectively since 2020. Patients were considered for inclusion if there was suspicion of any core clinical features of DLB, namely visual hallucinations, parkinsonism, cognitive fluctuations, or probable RBD. Some patients were included upon their first doctor’s visit, while other patients were retrospectively recruited at follow-up after receiving their diagnosis. Patients are excluded from Find-DLB if they terminated their evaluation before receiving a diagnosis at diagnostic rounds.

### Diagnostic procedure

Find-DLB being a naturalistic project, the procedure during diagnostic rounds follows the general Swedish guidelines of current clinical criteria. Patients with DLB were clinically diagnosed according to the consensus criteria available at the time of diagnosis [1, 19]. To ensure full adherence to current criteria, clinical diagnoses were retrospectively cross checked with the 2017 consensus criteria for DLB [1] by a senior physician (V.J.) and a neuropsychologist (S.G.) together. Unclear cases were fully reviewed by the senior physician (V.J.), who decided the final diagnosis based on all available information. Mild cognitive impairment (MCI) was diagnosed in accordance with the consensus criteria, if there is objective cognitive impairment on neuropsychological tests with relatively spared level of function in ADL [20]. Additional criteria were applied to establish the diagnosis of MCI due to PD (PD-MCI) [21] or MCI with Lewy bodies (MCI-LB) [22]. For patients who presented to the clinic with parkinsonism, the 1-year rule of onset between parkinsonism and dementia was applied to differentiate between DLB and PD [1]. Patients with two clinical diagnoses, i.e., both a diagnosis of DLB and AD, were included in the DLB group and reported as such for the purpose of the current study.

### Assessments

#### Clinical features

Clinical data regarding DLB core clinical features was collected through the electronic health records. Self-, caregiver-, and clinician-reported symptoms were all considered. Parkinsonism was coded as present if the records included descriptions of cardinal parkinsonian features of either bradykinesia, resting tremors, or rigidity. Visual hallucinations were considered present if there was mention of fully formed visual hallucinations. Fluctuations were coded as present if reported as either clear fluctuating cognitive impairment or alertness as well as passing episodes of confusion or delirium. Probable RBD was considered present if there were reported symptoms such as arm flailing, thrashing, punching, screaming or shouting, recurring nightmares, and falling out of bed during sleep time. If a clinical feature was negated, it was coded as not present. If there was no clear mention of a core clinical feature, it was considered missing data following a conservative criterion. Several patients reported unspecific symptoms or supportive clinical features such as visual illusions, presence phenomena, hyposmia, daytime sleepiness, delusions, and nightmares without other symptoms of RBD, which were not included in our current analysis due to not systematic documentation in health records.

#### Neuropsychological assessment

A subgroup of patients (*n* = 96, 67% of the total cohort, *n* = 143) underwent extended neuropsychological evaluations performed by a psychologist. The remaining 47 patients (33%) did not undergo neuropsychological assessment due to low performance on cognitive screening test, severe impairment in ADL, or frailty. Raw data from neuropsychological tests were available for 68 patients (48% of the total cohort) due to failure to submit raw scores in the electronic health records. Most patients underwent neuropsychological assessment within 6 months of their final diagnosis. However, six patients (6%) performed neuropsychological assessment more than 2 years before their dementia diagnosis. These assessments were carefully reviewed. Based on the available information, five of these patients were included in a group of patients without dementia, referred to below as the non-dementia (ND) group. The remaining patient was excluded from further analyses due to insufficient information.

All neuropsychological tests were administered at a single visit, extending for approximately 2–3 h. Since Find-DLB is a fully naturalistic cohort, there was variation in neuropsychological tests used across patients over time. Only tests with scores available for > 50% of the subgroup with neuropsychological assessment were included in this article, including the following:

*Verbal:* Wechsler Adult Intelligence Scale 4th edition (WAIS-IV), [23] and Wechsler Adult Intelligence Scale 3rd edition (WAIS-III) [24] Subtests: Information, Similarities.

*Memory:* Rey Auditory Verbal Learning Test (RAVLT) without distraction list [25]: total score over five learning trials and free delayed recall (30 min after learning trials); Rey Complex Figure Test (RCFT), immediate recall.

*Visuospatial:* WAIS-IV and WAIS-III Subtests: Block design, RCFT [26]: copy trial.

*Executive/attention/processing speed:* WAIS-IV and WAIS-III Subtests: Digit Span, Coding, Arithmetic, Matrix reasoning; Trail Making Test A and Trail Making Test B [27]: time to complete; Delis–Kaplan Executive Function System (D-KEFS) Trail Making Test 2 and 4 [28]: standardized score for time to complete.

Neuropsychological raw data was standardized to z-scores using normative data from regression-based norms used in the specialized cognitive clinic, stratified for age, education, and sex [29]. For WAIS and D-KEFS subtests, age-appropriate scaled scores were used.

A total of 57 patients performed subtests from WAIS-IV and 7 patients completed subtests from WAIS-III instead. To facilitate overview of the cognitive functions and domains, corresponding tests from different versions were combined. Specifically, WAIS-III and WAIS-IV standardized scores were combined into single variables for each subtest. Time to complete Trail Making Test A and D-KEFS Trail Making Test 2, as well as Trail Making Test B and D-KEFS Trail Making Test 4, was standardized and combined into two variables: TMT Number sequencing and TMT Letter number switching. For WAIS subtests, strong correlations between WAIS-III and WAIS-IV versions of the subtests have been previously reported, motivating their combination [23]. For the Trail Making Test, the placement, number of stimuli, and size of the paper differ, while the tasks are identical and can, therefore, be combined.

After standardization and combination of tests, scores were considered impaired if z-score was ≤ -1.5 SD, in accordance with established clinical criteria [30]. Discontinuation of a test due to the patient being unable to complete it was considered an impaired test: WAIS-IV Block design was discontinued for one patient, Trail Making Test B for nine patients, D-KEFS Trail Making Test 4 for two patients, and RCFT copy for three patients.

#### Biomarkers

Cerebrospinal fluid (CSF) biomarker status for amyloid-beta 1–42 and phosphorylated tau 181 (p-tau) was coded as positive or negative if below or above the center-specific cutoff. Since data was collected over a large time span, methods and cutoffs have changed. For clinical usefulness and interpretability, data is presented as positive or negative. The applied cutoffs are described elsewhere [31–33]. Dopamine transporter scan (DaT-Scan) was coded as positive or negative based on the report from the nuclear medicine department. DaT-Scan in one patient was deemed inconclusive, and it was excluded from the analysis of group differences. The current article focuses on introducing the Find-DLB cohort and describing the naturalistic diagnostic procedure. Further data beyond the availability of MRI, EEG, and FDG-PET will be presented in future modality-specific publications.

### Statistics

Descriptive data is presented as count and percentage or mean and standard deviation. *T* test or ANOVA-test with paired post hoc analyses with the Tukey adjustment was performed on continuous variables. *χ*^2^-tests were used for categorical variables, and Fisher–Freeman–Halton test was applied when any cell count was < 5, with paired post hoc Fisher exact tests. Significance level was pre-determined at *p* ≤ 0.05 in all analyses.

Additionally, we performed a random forest classification model to discriminate DLB from non-DLB patients (outcome variable). As predictors, we included availability (yes vs. no) of all core clinical features, MRI, EEG, FDG-PET, DaT-Scan, and amyloid-beta 1–42 and p-tau in CSF. We built 5000 trees with two predictors tested at each split, and balanced case sampling as the DLB and non-DLB groups had different sizes. The model´s classification performance was assessed through the estimate of the out of the box error. The contribution of each predictor to the model was represented by their proportion of importance (*Imp*).

Years of education were missing for four patients who underwent neuropsychological evaluation. The regression-based norms require years of education to calculate individual predicted values. Out of these four patients, two had information on occupation, and an estimated level of education was derived from the lowest level needed for the registered occupation within the Swedish educational system. For the other two patients, there was insufficient information, so that predicted values of education were estimated based on a linear regression model including the raw score of information from WAIS-IV as a common proxy of educational attainment [34, 35] along with age, sex, and diagnosis (i.e., DLB, PDD, or MCI). MMSE was missing for 39 patients who instead had performed the MoCA test. An estimated MMSE score was calculated from MoCA scores based on a conversion table published elsewhere [36].

## Results

### Whole find-DLB cohort

One forty four patients were eligible for inclusion in the Find-DLB cohort. One patient was excluded due to termination of the evaluation prior to a diagnosis. Table 1 shows that, out of the remaining 143 patients, 88 patients received a final clinical diagnosis of DLB (83 probable DLB and 5 possible DLB), with 8 of them receiving a clinical diagnosis of both DLB and AD. Out of the remaining patients, 35 were diagnosed with PDD; 2 patients were diagnosed with AD with atypical presentation such as prominent visuospatial impairment and neuropsychiatric symptoms; and 4 patients were included in the “Other dementias” group, which included corticobasal degeneration and dementia not otherwise specified. Furthermore, 14 patients received a clinical diagnosis of MCI (8 PD-MCI, 2 MCI-LB, and 4 had an MCI diagnosis without any specified cause) and were included in the non-dementia (ND) group. The two AD patients and the “Other dementias” group were excluded from further analysis due to small group sizes.

**Table 1:** Demographics and clinical features, whole cohort (*N* = 143)

Variable	Mean (SD), or count (%)	*N*
Age	70.8 (7.5)	142
Sex (male)	102 (71%)	143
Years of education	12.3 (3.4)	119
MMSE	24.1 (3.7)	142
Parkinsonism	120 (87%)	138
Visual hallucinations	87 (65%)	134
Probable RBD	55 (47%)	118
Cognitive fluctuations	50 (52%)	97
Final clinical diagnosis		143
DLB	88 (61.5%)	
Probable DLB	83 (58%)	
Possible DLB	5 (3.5%)	
PDD	35 (24.5%)	
Atypical AD	2 (1.4%)	
Other dementias*	4 (2.8%)	
MCI	14 (9.8%)	
MCI-LB	2 (1.4%)	
PD-MCI	8 (5.6%)	
MCI, unspecified	4 (2.8%)	

*Corticobasal degeneration and non-specified dementia. *MMSE* Mini Mental State Examination, *RBD* REM sleep behavior disorder

### Demographic and clinical characteristics and biomarkers of the whole Find-DLB cohort (*N* = 143)

Table 1 shows the demographic characteristics and presence of core clinical features within the whole cohort. The mean age at final diagnosis was 70.8 years, ranging from 52 to 88 years. Approximately, 71% were male and the mean years of education was 12.3. The mean MMSE score was 24.1, ranging from 11 to 30. Parkinsonism was the most frequent core clinical feature, with 87% of the patients experiencing at least one cardinal feature of parkinsonism. 65% experienced visual hallucinations, 52% had cognitive fluctuations, and 47% had symptoms of probable RBD. In this cohort, cognitive fluctuations was the core feature least frequently screened for and reported in health records, with data available only for 68% of the cohort.

Table 2 shows availability of different modalities and biomarkers in the cohort. 73% of the whole cohort completed a brain MRI and 74% an EEG examination, while a smaller proportion had an FDG-PET scan (38%). 50% of the patients completed a DaT-Scan and 93% of those had a result consistent with reduced dopamine transporter uptake in basal ganglia. One patient had an inconclusive DaT-Scan result according to the clinical report. Amyloid positivity in CSF was more common (20%) than p-tau positivity (8%).

**Table 2:** Available modalities and biomarkers in the whole Find-DLB cohort (*n* = 143)

Variable	Count	Positive	Negative
MRI	105 (73%)		
EEG	106 (74%)		
Neuropsychology	96 (67%)		
FDG-PET	54 (38%)		
DaT-Scan	72* (50%)	67 (93%)	4 (6%)
CSF amyloid-β 1–42	112 (78%)	22 (20%)	90 (80%)
CSF p-tau	111 (78%)	9 (8%)	102 (92%)

*Out of the 72 patients assessed with a DaT-Scan, *n* = 1 had an inconclusive result according to the clinical report. *MRI* brain magnetic resonance imaging, *EEG* electroencephalography, *FDG-PET* fluorodeoxyglucose positron emission tomography, *DaT-Scan* dopamine transporter scan, *CSF amyloid-β 1–42* cerebrospinal fluid amyloid beta 1–42, *CSF p-tau* cerebrospinal fluid phospholyrated tau

### Demographic and clinical characteristics and biomarkers by diagnostic group (DLB, PDD, and ND)

Table 3 shows the main demographic characteristics and biomarker data for DLB, PDD, and ND (the non-dementia group, composed of MCI patients). The DLB group was statistically significantly older compared to the ND group. There were no significant group differences in sex distribution or years of education. As expected, ND performed significantly better on MMSE compared to both DLB and PDD. Parkinsonism was the most frequent core clinical feature for all diagnostic groups with 100% of PD patients, almost 90% of DLB patients, and 67% of ND patients experiencing parkinsonism. Statistical testing shows that parkinsonism was significantly more common in PDD compared to ND. Both DLB (69%) and PDD (67%) had a higher frequency of visual hallucinations compared to ND (20%), but there were no significant group differences regarding probable RBD or cognitive fluctuations. In the smaller subsample with available CSF biomarkers and DaT-Scan, 27% of DLB participants were amyloid-positive in CSF, which was significantly more frequent than in PDD where only 4% were amyloid positive. There were no group differences regarding positivity on DaT-Scan or P-tau in CSF. DaT-Scan was only performed for 51% of all participants within these diagnostic groups. For the remaining participants, DaT-Scan status is unknown.

**Table 3:** Group comparison of demographics and biomarker status of available data

Variable	DLB = 88	*N*	PDD = 35	*N*	ND = 14	*N*	*p* value
Age, years	72.5 (7.0)	87	69.4 (7.6)	35	66.6 (6.9)	14	**0.005** a
Sex, male	62 (71%)	88	24 (69%)	35	11 (79%)	14	0.846
Education, years	12.0 (3.2)	77	12.4 (3.5)	25	14.0 (4.4)	10	0.226
MMSE, total score	23.6 (3.9)	87	24.0 (3.5)	35	26.9 (2.6)	14	**0.010** a,b
Parkinsonism, presence	77 (90%)	86	34 (100%)	34	8 (67%)	12	**0.005** b
Hallucinations, presence	59 (69%)	85	22 (67%)	33	2 (20%)	10	**0.010** a,b
Probable RBD, presence	37 (46%)	80	16 (57%)	28	1 (14%)	7	0.134
Fluctuations, presence	41 (57%)	72	6 (38%)	16	2 (40%)	5	0.291
CSF amyloid-β 1–42, positive	18 (27%)	68	1 (4%)	27	2 (18%)	11	**0.025** c
CSF p-tau, positive	5 (7%)	68	2 (8%)	26	1 (9%)	11	1.000
DaT-Scan, positive	46 (96%)	48	16 (94%)	17	4 (80%)	5	0.215

Mean (standard deviation) and count (percentage) are reported as detailed in the table. a) DLB vs ND. b) PDD vs ND. c) DLB vs PDD

*DLB* dementia with Lewy bodies, *PDD* Parkinson’s disease dementia, *ND* non-dementia group (composed of MCI patients), *MMSE* Mini Mental State Examination, *RBD* REM sleep behavior disorder, *Pos DaT-Scan* positive dopamine transporter scan, *CSF amyloid-β 1–42* cerebrospinal fluid amyloid beta 1–42, *CSF p-tau* cerebrospinal fluid phospholyrated tau

### Re-diagnosis of non-DLB patients for the formal fulfillment of ‘McKeith criteria’

In addition to the analysis by diagnostic group according to the final clinical diagnosis reported above, we wanted to gain additional information about the patients that received a non-DLB diagnosis. A total of 55 patients presenting at our clinic with clinical features of DLB received a final clinical diagnosis other than DLB, as detailed in Table 1. The random forest model to discriminate these 55 non-DLB patients from the 88 patients diagnosed with DLB showed that availability of data about cognitive fluctuations (*Imp* = 72.8) and FDG-PET (*Imp* = 42.4) were important to correctly classify patients as DLB, followed by RBD (*Imp* = 39.2), EEG (*Imp* = 33.2) and DaT-Scan (*Imp* = 15.5) (Supplementary Table 1 for full results of this model).

Furthermore, we assessed the frequency of core clinical features and indicative biomarkers along with the criterion of dementia in the non-DLB patients. Indicative biomarkers were solely based on DaT-Scan, as we did not have any data on polysomnography or ﻿MIBG myocardial scintigraphy in the Find-DLB cohort. This re-diagnosis process allowed us to quantify to what extent patients receiving a non-DLB clinical diagnosis do formally fulfill the ‘McKeith et al. (2017) criteria’ for DLB, thus documenting a problem that is common in the clinical reality (specificity of the ‘McKeith criteria’). For this analysis, we excluded the 14 patients with MCI as they did not fulfill the essential criterion of dementia, giving a total of 41 patients with dementia other than DLB. Supplementary Table 2 shows the frequency of a probable or possible diagnosis of DLB. Among the 41 non-DLB patients with dementia, 33 (80.5%) formally fulfilled a clinical diagnosis of probable DLB, 7 (17.1%) formally fulfilled a clinical diagnosis of possible DLB, and 1 (2.4%) did not fulfill a clinical diagnosis of DLB. Supplementary Table 3 shows the frequency and combinations of core clinical features for these 41 non-DLB patients with dementia, along with features in the DLB group for comparison. The most common single core clinical feature in non-DLB patients with dementia was parkinsonism, and the most common combination of features was parkinsonism with visual hallucinations or RBD, or a combination of the three. The findings were qualitatively similar in the DLB group, although combinations with cognitive fluctuations were less common in non-DLB patients.

### Neuropsychological profile

The subsample with neuropsychological data included 68 patients. Six patients completed the neuropsychological assessment > 2 years before the final diagnosis: one patient with DLB and five patients with PDD. The DLB patient had an MCI-LB diagnosis at previous visits. The five PDD patients had completed the assessment before converting to dementia. Therefore, they were grouped with the PD-MCI patients in the non-dementia (ND) group for the analyses reported in this section. To keep that group specific for PD, the remaining MCI patients were excluded from further analysis (two with MCI-LB and two MCI without specified cause). The final groups for comparing neuropsychological test performance were as follows, based on final clinical diagnoses (n = 63): DLB (n = 43), PDD (n = 9), and ND (n = 11).

Table 4 shows key demographic variables and neuropsychological test performance of DLB, PDD, and ND groups. DLB were older than PDD and ND, significantly so compared to PDD (p = 0.037). There were no significant differences in the years of education (p > 0.05).

**Table 4:** Neuropsychological test performance

Variable	DLB = 43	*N*	PDD = 9	*N*	ND = 11	*N*
Age at NP	71.4 (7.0)	43	64.8 (6.7)	9	68.7 (7.6)	11
Education	12.4 (3.2)	43	11.3 (3.4)	9	14.7 (3.6)	11
RAVLT learning	17 (44%)	39	1 (17%)	6	0 (0%)	10
RAVLT 30 min	17 (44%)	39	3 (38%)	8	1 (10%)	10
RCFT copy	21 (66%)	32	3 (50%)	6	3 (33%)	9
RCFT recall	20 (69%)	29	3 (50%)	6	3 (30%)	10
Information	3 (8%)	37	2 (29%)	7	0 (0%)	8
Similarities	10 (27%)	37	1 (13%)	8	0 (0%)	9
Block design	24 (59%)	41	3 (43%)	7	1 (10%)	10
Matrix reasoning	7 (35%)	20	2 (50%)	4	1 (11%)	9
Arithmetic	7 (32%)	22	2 (40%)	5	1 (14%)	7
Digit span	10 (25%)	40	1 (17%)	6	0 (0%)	9
Coding	17 (53%)	32	3 (43%)	7	1 (13%)	8
TMT numbers	19 (76%)	25	3 (75%)	4	1 (25%)	4
TMT Let/num	17 (77%)	22	4 (100%)	4	2 (40%)	5

Data is presented as count and percentage with impaired performance on each neuropsychological test, stratified by diagnostic group

*DLB* dementia with Lewy bodies, *PDD* Parkinson’s disease dementia, *ND* non-dementia group (composed of patients with Parkinson’s disease), *NP* neuropsychological assessment, *RAVLT* Rey Auditory Verbal Learning Test, *RCFT* Rey Complex Figure Test, *TMT numbers* Trail Making Test number sequencing, *TMT Letter/numbers* Trail Making Test letters and numbers switching

**Fig. 2:**
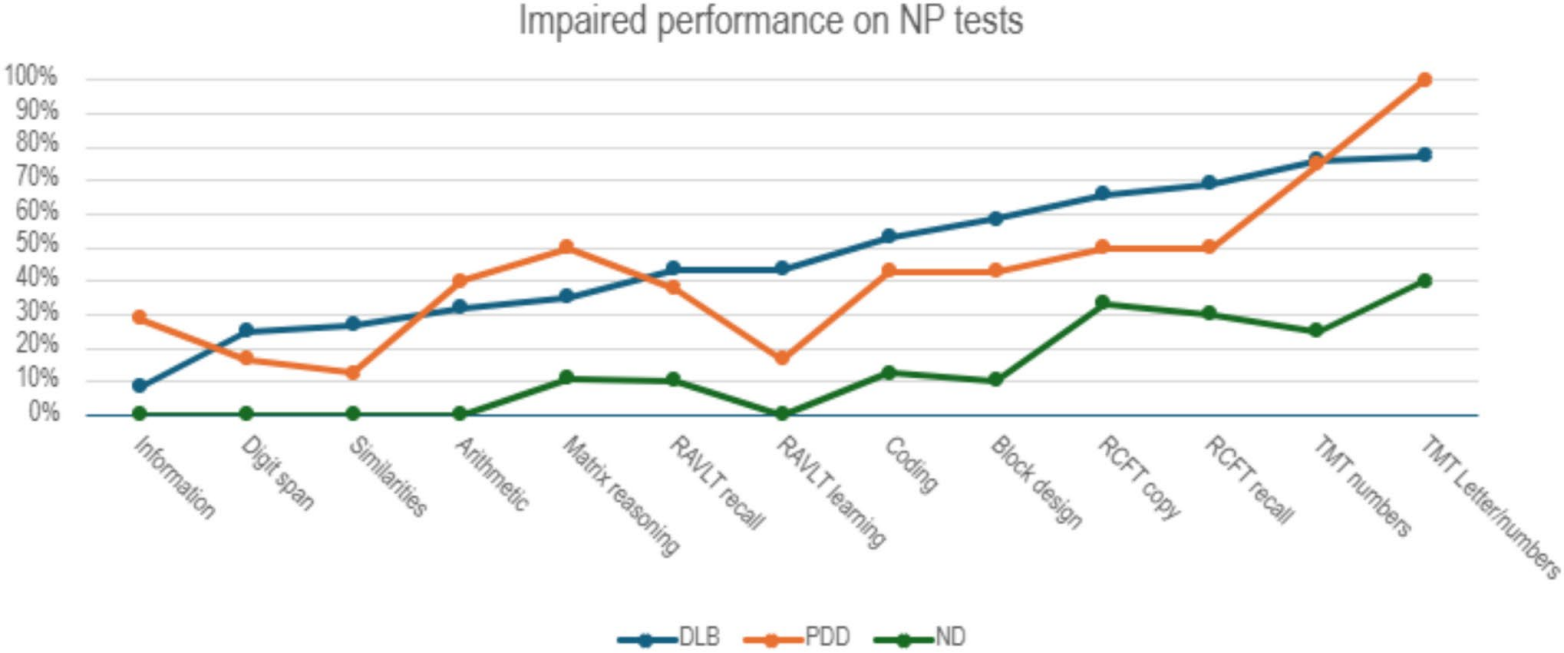
Neuropsychological profile of impairment. X-axis shows neuropsychological tests (NP), and Y-axis shows percentage of participants with impaired performance on each test. *DLB* dementia with Lewy bodies, *PDD* Parkinson’s disease dementia, *ND* non-dementia group (composed of patients with Parkinson’s disease), *RAVLT* Rey Auditory Verbal Learning Test, *RCFT* Rey Complex Figure Test, *TMT numbers* Trail Making Test number sequencing, *TMT Letter/numbers* Trail Making Test letters and numbers switching

Neuropsychological test performance was classified into normal or impaired and percentage of impairment was reported (Table 4) and plotted to build impairment profiles (Fig. 2). Due to the small sample size in the neuropsychological subsample, we first inspected the percentage of impairment qualitatively. We observed that DLB generally had higher percentages of impairment compared to both ND and PDD. As expected, ND overall had the lowest proportion of impaired scores. Ordered by frequency of impairment, Fig. 2 shows that the most frequently impaired cognitive domains in DLB were visual attention and processing speed. More than 75% of DLB patients were impaired on trail making tasks, both number sequencing and number–letter switching. An even higher proportion of impairment was seen in PDD, where 100% were impaired on number–letter switching task. Out of all cognitive tests, the number–letter switching task showed the highest level of impaired performances across all three groups, including ND (see Table 4 for full details on percentage of impairment in each diagnostic group).

Next, more than 50% of the DLB group had impaired performance on mental processing speed (Coding), visuospatial constructive tasks (both Block design and RCFT copy), and visual memory (RCFT immediate recall). RCFT copy was impaired in 66% of DLB patients, and free recall was impaired in 69%. In comparison, 50% of PDD patients had impaired performance on these tests.

Verbal learning and delayed recall (RAVLT learning and delayed recall) were impaired in 44% of DLB patients, while ND generally performed within normal range on both learning and delayed recall. PDD had a higher proportion of impaired performance on delayed recall (38%) compared to learning (17%), but it should be noted that the learning trial was missing for two PDD participants.

Semantic memory and general knowledge (Information), auditive short-term memory and working memory (Digit span), abstract verbal thinking (Similarities), and arithmetic ability (Arithmetic) were impaired in less than a third of DLB patients, and logical thinking (Matrix reasoning) was impaired in 35%, meaning that a significant proportion of patients performed within the normal range on these cognitive tests. In comparison, 40% of PDD participants were impaired on arithmetic, and 50% on matrix reasoning.

For completeness of information, we followed visual inspection with statistical group comparisons. Despite the reduced statistical power in the smaller subsample with neuropsychological data, we obtained a statistically significant difference between DLB and ND on Block design (*p* = 0.011) and verbal learning on RAVLT (*p* = 0.009), which were both more frequently impaired in DLB. There were no other significant group differences on the other reported neuropsychological tests (*p* > 0.05).

## Discussion

Our main goal was to describe a naturalistic cohort of patients presenting with clinical features of DLB to a specialized cognitive clinic. We describe the diagnostic procedure and report baseline demographic and clinical data and neuropsychological profiles. 143 patients were included. 88 patients had a final clinical diagnosis of DLB, while 35 had PDD, 14 MCI, and 6 patients had other types of dementia. There were no statistically significant differences in frequency of any core clinical features between DLB and PDD. However, amyloid positivity as measured in CSF was significantly more frequent in DLB than in PDD. The non-dementia group was heterogeneous and included participants with MCI-PD, MCI-LB, and MCI without specified cause. Visual hallucinations were less frequent in ND participants compared to both DLB and PDD.

### Clinical diagnostic challenges

Out of 143 patients presenting with core clinical features of DLB, 88 fulfilled criteria for probable or possible DLB. A majority of the non-DLB patients had PD-related cognitive impairment, i.e., PDD or PD-MCI. While most of these PD patients had been diagnosed with PD prior to referral, there may still be diagnostic challenges. The average time from PD diagnosis to PDD onset has been reported to be approximately 10 years [37]. An aggressive and faster progression of cognitive decline may raise suspicion of DLB. Some patients or caregivers retrospectively report the onset of cognitive impairment to have been simultaneous or prior to the parkinsonian features, and others receive a diagnosis of dementia closely after the 1-year mark, making it difficult to determine the exact time of dementia onset. As we carefully reviewed cases retrospectively to confirm the final diagnosis, we strictly employed the 1-year rule to properly differentiate DLB from PDD [1].

Additionally, the clinical separation of DLB from AD proved challenging. While the proportion of patients receiving a clinical diagnosis of AD was only around 1% in this cohort, eight patients had been given a clinical diagnosis of both DLB and AD. When inspecting health records, we observed that some patients had received a previous clinical AD diagnosis which was later converted to DLB when core clinical features were unveiled or became more prominent. Previous studies have reported that around 50% of DLB patients show evidence for amyloid-related pathology [5, 7]. In our cohort, only 27% of DLB patients were amyloid positive in CSF. This could possibly be explained by DLB patients being misdiagnosed with AD and therefore not included in this cohort. The first step of the diagnostic workup in our cognitive clinic usually includes a lumbar puncture along clinical assessments, and additional tests are added if needed in the second step. This setup could favor a diagnosis of AD if AD biomarkers in CSF are positive, in line with previous reports [8].

In our clinical experience, the supportive biomarkers that help the most in guiding the diagnostic process in unclear cases are the positive cingulate island sign on FDG-PET and posterior slow wave activity on EEG, in accordance with the consensus criteria [1]. We supported this clinical observation with a classification random forest model performed on the available data in this cohort. This model confirmed that the availability of FDG-PET and EEG was important to correctly classify patients with DLB, along with the availability of data on cognitive fluctuations, probable RBD, and DaT-Scan.

### Frequency of the core clinical features

Overall, parkinsonism was the most common core clinical feature across all diagnostic groups in the Find-DLB cohort. 90% of DLB patients had at least one cardinal feature of spontaneous parkinsonism. While we did not stratify for different features of parkinsonism, previous studies have found rigidity and bradykinesia in up to 85% of DLB patients, with lower prevalence of resting tremors [38]. Visual hallucinations were present in almost 70% of DLB patients, in line with previous reports [14, 38, 39]. Probable RBD was present only in 46% of the DLB patients and was the least common out of the core features. This is lower than in other DLB cohorts, where RDB has been reported in about 76% of neuropathologically confirmed cases [39]. The lower frequency of probable RBD in our cohort may thus be related to the lack of a bed partner to report RDB-related symptoms as well as the lack of polysomnography. Furthermore, the least documented symptom in health records in our cohort was cognitive fluctuations, which has long been considered a difficult symptom to capture [40]. Cognitive fluctuations may be difficult to define and explain to the patient and caregiver. The prospective collection of cases in the Find-DLB cohort has thus included validated questionnaires for probable RBD [41], and a clinical rating scale for fluctuations [42]. Hence, in the future we will be able to compare the prospective rates with those in the current report based mostly on retrospective cases.

### The “cognitive profile” of impairment in DLB

A majority of DLB patients were impaired on visuospatial constructive tasks, visual memory, and processing speed tasks such as digit symbol and trail making tests. This is in line with previous research, which has found the cognitive profile in DLB to show a larger proportion of visual perceptual, attentional, and executive impairment compared to other cognitive domains [1]. Visual memory impairment in DLB has been shown to be comparable to that of AD [43, 44]. It is possible that this reflects the visual processing deficit common in DLB influencing the memory encoding process, rather than representing a genuine memory impairment.

Interestingly, 44% of DLB patients had impaired verbal learning and delayed free recall. While the memory deficit in DLB has been hypothesized to be partially related to encoding and retrieval deficits rather than memory storage, previous studies found that a relevant portion of DLB patients may have impaired verbal memory storage and consolidation, even at MCI-LB stages [43, 45]. A limitation of our Find-DLB cohort is the lack of memory recognition subtasks, which would help clarify whether our memory finding is related to encoding and retrieval or memory storage and consolidation.

WAIS-subtests Digit span and Arithmetic were impaired in less than a third of DLB patients, meaning that a majority performed within normal range on tasks traditionally considered as working memory processes. Previous research has found working memory to be impaired in DLB, and more severely impaired than in AD [44, 46]. WAIS-subtest Similarities, measuring abstract thinking and considered partly executive in nature, was also preserved in a large proportion of DLB patients in our cohort. It should be noted that the lower proportion of impaired performances on some neuropsychological tests could be influenced by the selection process of the subgroup with neuropsychological assessment in our cohort. In our clinic, patients with severe impairment on cognitive screening may not undergo neuropsychological assessment and, thus, would not be included in these analyses.

When comparing DLB with PDD, visual inspection suggested similar frequencies of impairment on tests such as complex figure copy and recall, as well as trail making tasks. However, PDD had a lower proportion of impaired patients on several neuropsychological tests. Specifically, impairment in verbal learning in PDD was similar to ND and qualitatively milder than that in patients with DLB. Some previous studies have reported differences in memory impairment between PDD and DLB with a trend towards lower learning and memory retention in DLB, while other studies did not find any significant differences [9, 10]. In our study, the qualitative differences between DLB and PDD in cognitive performance are not explained by differences in global cognitive performance as reflected by the MMSE. As expected, ND generally had a smaller proportion of impaired performances on most tests, compared to both DLB and PDD. Despite the limited statistical power in the subsample with neuropsychological data available, we followed visual inspection of percentage of impairment with formal statistical testing for completeness of information. We found that verbal learning from RAVLT and Block design from WAIS were statistically significantly more frequently impaired in DLB than in ND. We did not capture any other statistically significant results, likely because of the small sample size of the PDD and ND groups.

In summary, cognitive impairment in our DLB patients was primarily characterized by impairment in visuospatial constructive tasks, visual memory, visual attention and processing speed, as well as executive functions as measured by task switching. The profile is in line with previous reports of commonly affected domains in DLB [1]. Auditory short-term and working memory tasks were less frequently impaired in our DLB group.

### Limitations

This study has some limitations. Firstly, due to the clinical nature of the data, some variables were incomplete and the diagnostic battery for each patient may differ. This may increase the risk of bias, such as within the context of neuropsychological assessment—patients with severe cognitive impairment may be less likely to undergo full neuropsychological evaluation. However, the bias may be less than in research-oriented cohorts that often recruit DLB patients at mild to moderate stages. Secondly, supportive clinical features were not systematically collected previously in our clinic and therefore not included in the current study. Thirdly, the small sizes of PDD and ND groups likely limited the possibility to obtain more statistically significant differences in neuropsychological variables. We circumvented this by using normative data and reporting frequencies of impairment across tests as the main analyses, while statistical comparisons were explorative and reported for completeness of information. The selection of norms was made carefully since norms may affect the interpretation of what constitutes an impairment. Further, the chosen norms are applied in the clinic, which improves generalizability to a clinical context. All norms were age stratified and occasionally accounted for sex and education, all factors known to influence performance on some cognitive tests. Lastly, we included patients with possible DLB as well as patients with a concurrent clinical diagnosis of DLB and AD, since our aim was to present a realistic and naturalistic clinical cohort. However, whether the frequency of cognitive impairment differs across probable DLB, possible DLB, and DLB cases with a clinical phenotype of mixed AD and DLB should be investigated further.

## Conclusion

This article describes the Find-DLB cohort, a cohort of patients presenting with clinical features of DLB to our specialized cognitive clinic, in a naturalistic manner. Our study shows that even with access to several diagnostic modalities, diagnosing DLB is challenging within the clinical context. Overall, the diagnostic pathway may increase the risk of underdiagnosing DLB in the case of positive AD biomarkers, even in the presence of core clinical features of DLB. The access to biomarkers such as EEG and FDG-PET may increase the certainty of a diagnostic decision but does not provide indicative biomarker information, and in the current cohort the only indicative biomarker accessible was DaT-Scan. The diagnostic process from first visit with a primary care physician to a DLB diagnosis is time and resource consuming, for the health care system, patient, and caregiver. More research should investigate this process and identify the factors susceptible to improvement, and clinicians should take care not to oversee or miss clinical features of DLB. The core clinical features are the foundation of the diagnostic process in DLB, and while parkinsonism and visual hallucinations may be more obvious during the clinical assessment, cognitive fluctuations and RBD can be more dubious, which was reflected in their low availability in this cohort. Moving forward, the evaluation of core and supportive clinical features would benefit from systematic data collection using validated clinical rating instruments. The recent emergence of seed amplification assays for synucleinopathy are expected to improve DLB diagnosis substantially, but there are currently several challenges for their clinical implementation [47].

## Supplementary Information

Below is the link to the electronic supplementary material.

Supplementary file1 (DOCX 22 KB)

## Data Availability

The data that support the findings of this study are not publicly available due to privacy and are protected by a pseudonymization protocol. Data may be available upon reasonable request from the corresponding author if legal and ethical requirements are met.

## Abbreviations

AD: Alzheimer’s disease
ADL: Activities of daily living
CSF: Cerebrospinal fluid
CT: Computer tomography
D-KEFS: Delis–Kaplan executive function system
DaT-Scan: Dopamine transporter scan
DLB: Dementia with Lewy bodies
EEG: Electroencephalography
EOD: Early-onset dementia
FDG: Fluorodeoxyglucose
ICD-10: International Statistical Classification of Diseases and Related Health Problems 10th Revision
MCI: Mild cognitive impairment
MCI-LB: MCI with Lewy bodies
MMSE: Mini Mental State Examination
MoCA: Montreal Cognitive Assessment
MRI: Magnetic resonance imaging
ND: Non-dementia
PDD: Parkinson’s disease with dementia
PD: Parkinson’s disease
PD-MCI: MCI due to PD
PET: Positron emission tomography
p-tau: Phosphorylated tau
RAVLT: Rey Auditory Verbal Learning Test
RBD: REM sleep behavior disorder
RCFT: Rey Complex Figure Test
TMT: Trail Making Test
WAIS: Wechsler Adult Intelligence Scale
