# Supplementary material for: Find-DLB: a naturalistic cohort of patients presenting with clinical features of dementia with Lewy bodies to a specialized cognitive clinic
Source: Eur Geriatr Med. 2025 Dec 8;17(1):309–21. doi: 10.1007/s41999-025-01372-z (PMC12946240; doi:10.1007/s41999-025-01372-z)
Supplement: Supplementary file 1 — Supplementary file1 (DOCX 22 KB) [file 41999_2025_1372_MOESM1_ESM.docx]

**SUPPLEMENTARY MATERIAL**

**Supplementary Table 1.** Random forest classification model for the discrimination of patients with DLB from non-DLB patients based on availability of data.

| **Overall classification performance**  **(N = 143, where 88 DLB and 55 non-DLB)** | | |
| --- | --- | --- |
| OOB error rate = 30.8%  Error by change is 50% | | |
| **Predictors**  **(ordered by Imp)** | **Proportion of importance (Imp)** | **Mean Decrease Gini** |
| Cognitive fluctuations | 72.8 | 7.850 |
| FDG-PET | 42.4 | 4.607 |
| Probable RBD | 39.2 | 4.645 |
| EEG | 33.2 | 3.676 |
| DaT-Scan | 15.5 | 2.526 |
| **Predictors not retained in the model** |  |  |
| Parkinsonism | - | - |
| Visual hallucinations | - | - |
| MRI | - | - |
| CSF Amyloid-β 1-42 | - | - |
| CSF p-tau | - | - |

*Predictors not retained in the model (step-wise backwards) do not contribute to the classification and, therefore, Imp and Mean Decrease Gini are not reported as reflected by the dash symbol. Abbreviations: MRI = Brain Magnetic Resonance Imaging. EEG = Electroencephalography. FDG-PET = Fluorodeoxyglucose Positron Emission Tomography. DaT-Scan = Dopamine Transporter Scan. CSF Amyloid-β 1-42 = Cerebrospinal Fluid Amyloid beta 1-42. CSF p-tau = Cerebrospinal Fluid Phospholyrated Tau. RBD = REM sleep Behavior Disorder. Imp = Proportion of Importance.*

**Supplementary Table 2.** Re-diagnosis of non-DLB patients for the formal fulfillment of ‘McKeith criteria’.

| **Non-DLB patients with dementia** | **Count** |
| --- | --- |
| ***Final diagnosis in electronic health records*** | **41** |
| PDD | 35 |
| Atypical AD | 2 |
| Other dementias (CBD, unspecified) | 4 |
|  |  |
| ***Re-diagnosis*** |  |
| **Fulfil formal criteria for probable DLB** | **33** |
| 2 or more core features (+- positive DaT-Scan) | 29 |
| 1 core feature and positive DaT-Scan | 4 |
|  |  |
| **Fulfil formal criteria for possible DLB** | **7** |
| 1 core feature | 6 |
| 0 core features and positive Dat-Scan | 1 |
|  |  |
| **No fulfilment of criteria for DLB** | **1** |
| 0 core features and negative Dat-Scan | 1 |

*DLB = Dementia with Lewy Bodies; PDD = Parkinson´s Disease with Dementia; AD = Alzheimer´s disease; CBD = Corticobasal Degeneration.*

**Supplementary Table 3.** Frequency and combinations of core clinical features in non-DLB patients with dementia compared with patients with DLB.

|  | **DLB**  **Count (%)** | **Non-DLB dementia**  **Count (%)** |
| --- | --- | --- |
| **No features** | **2 (2%)** | **2 (5%)** |
| No features | 1 (1%) | 2 (5%) |
| Missing features | 1 (1%) | 0 (0%) |
|  |  |  |
| **1 feature** | **18 (20%)** | **10 (24%)** |
| PK | 11 (13%) | 8 (20%) |
| CF | 1 (1%) | 0 (0%) |
| VH | 4 (4%) | 2 (4%) |
| RBD | 2 (2%) | 0 (0%) |
|  |  |  |
| **2 features** | **19 (22%)** | **14 (34%)** |
| PK/CF | 5 (6%) | 0 (0%) |
| PK/VH | 8 (9%) | 7 (17%) |
| PK/RBD | 4 (5%) | 5 (12%) |
| CF/VH | 1 (1%) | 0 (0%) |
| CF/RBD | 0 (0%) | 0 (0%) |
| VH/RBD | 1 (1%) | 2 (5%) |
|  |  |  |
| **3 features** | **38 (43%)** | **13 (32%)** |
| PK/CF/VH | 19 (22%) | 5 (12%) |
| PK/CF/RBD | 4 (4%) | 0 (0%) |
| PK/VH/RBD | 15 (17%) | 8 (20%) |
| CF/VH/RBD | 0 (0%) | 0 (0%) |
|  |  |  |
| **4 features** | **11 (13%)** | **2 (5%)** |
| PK/CF/VH/RBD | 11 (13%) | 2 (5%) |

*PK = Parkinsonism; CF = Cognitive Fluctuations; VH = Visual Hallucinations; RBD = REM sleep behavior disorder*
